# Exploring the effects of electrospun fiber surface nanotopography on neurite outgrowth and branching in neuron cultures

**DOI:** 10.1371/journal.pone.0211731

**Published:** 2019-02-04

**Authors:** Anthony R. D’Amato, Devan L. Puhl, Alexis M. Ziemba, Christopher D. L. Johnson, Janneke Doedee, Jonathan Bao, Ryan J. Gilbert

**Affiliations:** 1 Department of Biomedical Engineering, Rensselaer Polytechnic Institute, Troy, New York, United States of America; 2 Center for Biotechnology and Interdisciplinary Studies, Rensselaer Polytechnic Institute, Troy, New York,United States of America; The College of William and Mary, UNITED STATES

## Abstract

Three aligned, electrospun fiber scaffolds with unique surface features were created from poly-L-lactic acid (PLLA). Fibers without surface nanotopography (smooth fibers), fibers with surface divots (shallow pits), and fibers with surface pits (deeper pits) were fabricated, and fiber alignment, diameter, and density were characterized using scanning electron microscopy (SEM). Whole dorsal root ganglia (DRG) were isolated from rats and placed onto uncoated fibers or fibers coated with laminin. On uncoated fibers, neurite outgrowth was restricted by fibers displaying divoted or pitted nanotopography when compared to neurite outgrowth on smooth fibers. However, neurites extending from whole DRG cultured on laminin-coated fibers were not restricted by divoted or pitted surface nanotopography. Thus, neurites extending on laminin-coated fibers were able to extend long neurites even in the presence of surface divots or pits. To further explore this result, individual neurons isolated from dissociated DRG were seeded onto laminin-coated smooth, pitted, or divoted fibers. Interestingly, neurons on pitted or divoted fibers exhibited a 1.5-fold increase in total neurite length, and a 2.3 or 2.7-fold increase in neurite branching compared to neurons on smooth fibers, respectively. Based on these findings, we conclude that fiber roughness in the form of pits or divots can promote extension and branching of long neurites along aligned electrospun fibers in the presence of an extracellular matrix protein coating. Thus, aligned, electrospun fibers can be crafted to not only direct the extension of axons but to induce unique branching morphologies.

## Introduction

In 2017, 17,700 Americans sustained spinal cord injury [1]. The prevalence of peripheral nerve injury is more difficult to discern. A review by Wiberg and Terenghi in 2003 stated that 2.8% of all trauma patients sustain some form of peripheral nerve injury [2]. Aligned electrospun fibers are studied for neural engineering applications as a potential therapy to promote directed tissue regeneration and functional recovery following nerve injury [3–7]. The diameter and highly aligned organization of some electrospun fiber scaffolds provide contact guidance for cells in the nervous system [3,6]. Because electrospun fibers direct the extension of axons, recent studies have augmented fiber physical properties to stimulate faster axonal extension.

To determine if augmenting fiber physical properties (such as alignment, density, and diameter) enhances the potential of fibers as scaffolds for neural injury repair, several studies examined how these properties affected the rate and extent of neurite extension in culture or *in vivo*. Wang et al. observed longer neurite extension (40% longer neurites) when dorsal root ganglia (DRG) were cultured on fibers with a mean diameter of 759 nm compared to DRG cultured on fibers with a mean diameter of 293 nm [8]. The degree of fiber alignment also influences the direction and extent of axonal regeneration and neurite extension. Hurtado et al. implanted either aligned (97% of fibers aligned with 4° of mean fiber alignment) or randomly oriented electrospun fiber scaffolds into a fully transected rat spinal cord. After four weeks, nervous tissue regenerated nearly twice as far into the aligned fiber conduit compared to tissue in conduits with randomly oriented fibers [9]. The ability of aligned fibers to support directed neurite extension was also revealed from DRG [10], PC12 cells, and primary hippocampal neurons [11]. In these *in vitro* studies, aligned fibers enhanced the length of directed neurite extension compared to randomly oriented fibers. Regarding fiber density, Wang et al. showed that increasing the fiber collection density from 480 fibers/mm to 620 fibers/mm increased the density of neurites extending from DRG explants by 18% [12]. While fiber alignment, density, and diameter have been studied extensively for neural applications, only two studies to our knowledge have analyzed the effects of electrospun fiber surface nanotopography on neurons [11,13]. Neither of these studies, however, controlled for the aforementioned fiber properties to specifically isolate the effects of fiber surface nanotopography on neurons.

Aside from changing physical properties to improve the regenerative potential of electrospun fibers for neural injury repair, studies have also modified the chemistry of fibers to stimulate axonal regeneration. Researchers commonly incorporate laminin, an extracellular matrix glycoprotein found in the basal lamina of most tissues, into biomaterials to increase their regenerative potential, especially in neural engineering applications [14]. Xie et al. showed that coating electrospun fibers with laminin caused adhering DRG to extend neurites that better followed fiber orientation and avoided neurite outgrowth perpendicular to the fiber alignment, when compared to uncoated fibers [15]. Other studies have directly electrospun laminin into fibers and showed that these fibers support tissue regeneration *in vitro*, and in animal models of peripheral nerve injury [16–18]. To date, however, no study has explored the interplay between laminin coating and electrospun fiber surface nanotopography to increase the regenerative potential of electrospun fibers in neural engineering applications.

In this study, we fabricated three types of electrospun fibers, each with distinct surface nanotopography: 1) Smooth fibers with no surface depressions, 2) Pitted fibers with distinct, well-defined surface depressions, and 3) Divoted fibers with ill-defined surface depressions that create a roughened surface topography. Fiber surface nanotopography was created by changing the humidity of the electrospinning environment, and/or including a non-solvent of the electrospinning polymer (poly(L-lactic acid), PLLA) in the electrospinning solution. These methods of engineering electrospun fiber surface nanotopography are discussed in detail in previous laboratory publications where astrocyte and macrophage response to changes in fiber surface topography were examined [19–21]. Having already analyzed astrocyte and macrophage response to fibrous nanotopography, the emphasis of this study focused on understanding how the presence of nanotopography affected neurite extension.

After creating fibers with three distinct surface topographies, we characterized fiber physical properties (alignment, density, and diameter) using scanning electron microscopy (SEM) to ensure that there were no significant differences between the three fiber types, aside from differences in surface nanotopography. This was necessary so that we could explicitly investigate the effects of fiber nanotopography on neurite extension from whole DRG and dissociated neurons. Once the effects of fiber surface nanotopography on neurons was understood, we coated all three fiber types with laminin to explore the interplay between fiber surface nanotopography and the biochemical cues provided by laminin.

## Materials and methods

### PLLA film casting

All fibers used for neural cultures in this study were electrospun onto PLLA films. Films were necessary so fibers would not lift off of the cover slip after submersion in culture media. A 4% w/w solution of PLLA (LACTEL Absorbable Polymers, Birmingham, AL) in chloroform (Sigma, St. Louis, MO) was drop casted onto 15 x 15 mm glass cover slips (Knittel Glass, Brausenweig, Germany). Films were vacuum dried overnight to remove chloroform prior to electrospinning.

### Electrospinning

All electrospinning for this study was performed inside of a 35 x 36 in. dissipative PVC glove box (Terra Universal, Fullerton, CA). Prior to electrospinning, a humidifier outside of the box was used to humidify the electrospinning room while the glove box was open. Immediately before electrospinning, once the relative humidity in the room had equilibrated to the desired level, the glove box was closed to maintain the relative humidity during electrospinning. Tight control over humidity was imperative to reproducibly fabricate electrospun fibers with the desired surface nanotopography [19–21].

**Fig 1:**
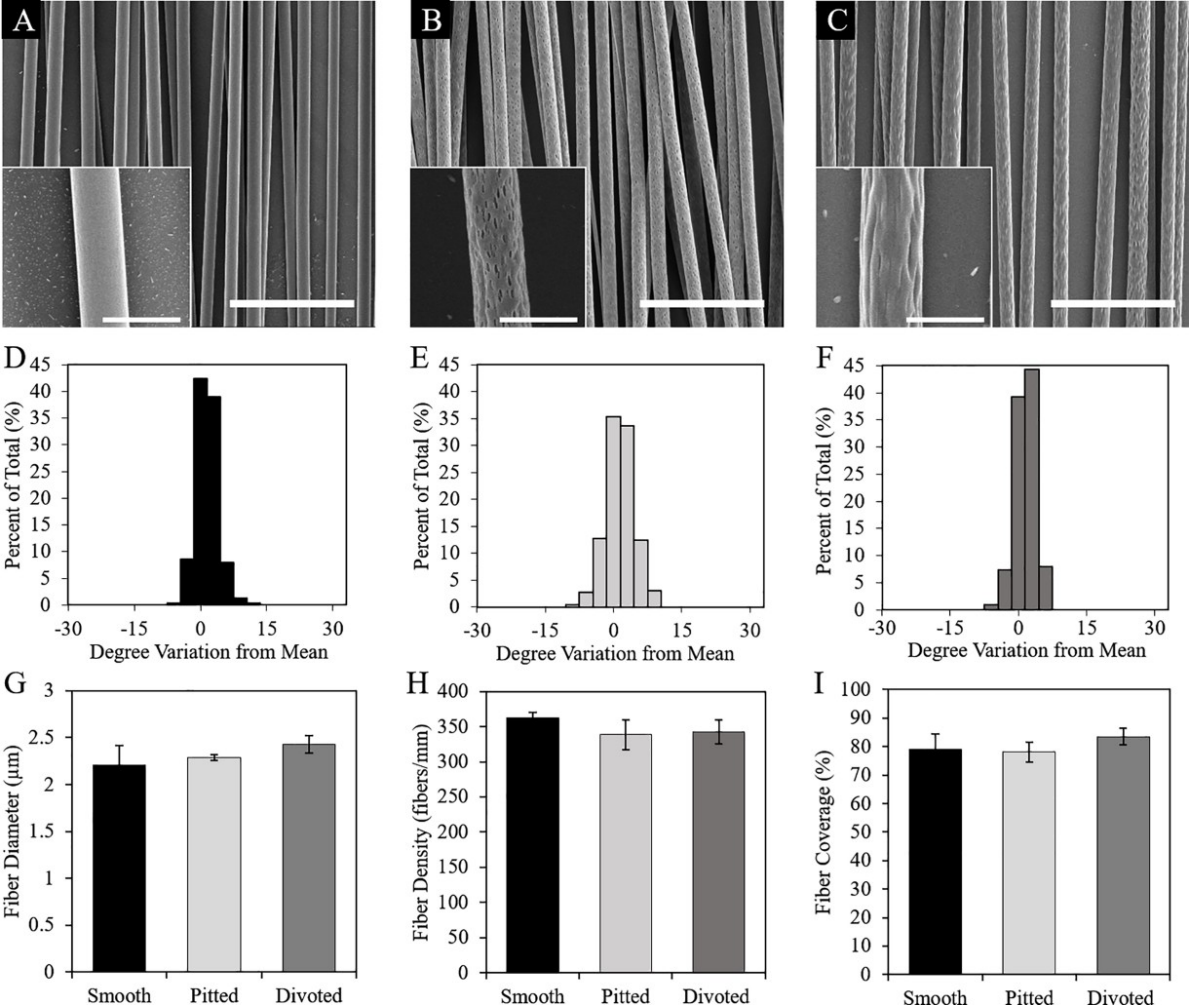
Fiber SEM and morphological characterization. (A-C) SEM images of smooth, pitted, and divoted fibers with inlaid close-ups to emphasize differences in surface nanotopography. Scale bars in main images are 20 μm, scale bars in inlaid images are 5 μm. (D-F) Fiber alignment was not statistically significant between fiber types. Histograms for smooth (D), pitted (E), and divoted (F) fibers represent the percentage of fibers within a certain angle of deviation from mean fiber alignment. Fiber diameter (G), collection density (H), and coverage (I) were also not statistically significant when compared between different fiber types. (Each type of fiber scaffold was fabricated in triplicate (N = 3), data are presented as mean ± standard deviation).

Inside of the glove box a syringe pump was affixed over a rotating, grounded aluminum mandrel (22 cm diameter, 1 cm thick). PLLA films on glass coverslips were attached to the collection mandrel with a piece of double sided tape. A 5 mL syringe (Becton Dickinson, Franklin Lakes, NJ) loaded with electrospinning solution was attached to a 22 gauge needle (Becton Dickinson), placed in the syringe pump, and connected to a Gamma High Voltage Power Supply (Model No. ES5OP-10W, State College, PA). Different electrospinning solutions (Table 1) were used to create three different types of fibers with either smooth, pitted, or divoted surface topographies (Fig 1A–1C). All fibers were electrospun in triplicate from three independently prepared polymer solutions (N = 3). Electrospinning solutions were prepared by combining PLLA, chloroform, and DMSO (for pitted fibers only) in a sealed glass vial, and stirring with a magnetic stir bar for three hours. While solution composition and relative humidity varied between fiber types, all fibers were electrospun for 10 minutes with a 15 kV applied voltage, 2 mL/h solution flow rate, 1000 rpm rotational mandrel speed (linear speed– 11.5 m/s), and 5 cm gap distance between the needle tip and mandrel. These electrospinning parameters produced a uniform monolayer of electrospun fibers.

**Table 1:** Electrospinning parameters.

Fiber Type	Polymer w/w% in Solvent	DMSO w/w % in Solvent	Relative Humidity during Electrospinning
**Smooth**	12	0%	≤ 21%
**Pitted**	12	1.80%	28–32%
**Divoted**	12	0%	28–32%

### Preparation of electrospun fibers for scanning electron microscopy (SEM) analysis

Electrospun fibers were imaged using SEM. Prior to SEM, coverslips with fibers were mounted onto aluminum SEM stubs (Ted Pella, Redding, CA) and sputter coated with a 1 nm layer of Gold/Palladium using a Technics V Sputter Coater (Anatech Ltd., Denver, CO). Fibers were then imaged at a low accelerating voltage (2–5 kV) using a FEI Versa 3D DualBeam SEM (Hillsboro, OR).

### Electrospun fiber morphological characterization

SEM images of electrospun fibers were analyzed using NIH FIJI software (Bethesda, MD) to characterize fiber physical properties including fiber alignment, diameter, and collection density. It was important to quantify and control for these physical properties to isolate the effects of fiber surface nanotopography in neuron cultures. All fiber characterization was conducted on a total of nine SEM images for each fiber type. Three independently fabricated fiber scaffolds were imaged via SEM (N = 3), and three SEM images were taken per scaffold to produce these nine images.

Fiber alignment was characterized by drawing a line parallel to each fiber in a given SEM image and calculating the mean angle of fiber orientation. The difference between each fiber’s angle of orientation and the mean angle of fiber alignment was calculated. Angles of deviation were binned and histograms were created depicting each fiber type’s overall alignment. To measure fiber diameter, a line was drawn spanning the width of each individual fiber in a given SEM image. The pixel length of each line was converted to microns using the SEM image’s scale bar. Lastly, to measure fiber collection density, all fibers in a given SEM image were counted and divided by the width of the image’s field of view to yield a value with units of fibers/mm. This value was then multiplied by the average diameter of each respective fiber type to yield a value describing the fiber coverage of a given scaffold.

### Electrospun fiber specific surface area characterization

We characterized the specific surface area of all three electrospun fibers types to determine if differences in specific surface area correlated with any observed differences in neuron culture metrics. Brunauer-Emmett-Teller (BET) surface area analysis was conducted using a Quantichrome Autosorb iQ (Model 6, Boynton Beach, FL), to quantify changes in the specific surface area (surface area per mass of fibers) of the three fiber types. Fiber specific surface area was measured using nitrogen gas physisorption at 77 K. Prior to surface area characterization, fibers were degassed in a vacuum oven at 40°C for 24 h. Relative pressures (P/P_0_) ranging from 0.05 to 0.3 were used to calculate surface area via BET using ASiQwin software (Version 5.00). The specific surface area of each electrospun fiber type was analyzed in triplicate from three independently fabricated fiber samples (N = 3). Each replicate consisted of approximately 400 mg of electrospun fibers fabricated from a single electrospinning solution. To produce this large amount of fibers, we increased our electrospinning collection time from 10 minutes to 45 minutes. Prior to BET analysis we conducted SEM on a fiber sample from each electrospinning batch to ensure that increasing the electrospinning collection time did not impact the fiber surface nanotopography or diameter. Fibers generated with this increased collection time were only used for BET surface area characterization, and not in any cell culture experiments.

### Dorsal root ganglion (DRG) isolation and culture

#### DRG isolation

DRG cultures are commonly used to understand the effects of neural engineering approaches or pharmaceutical interventions [22,23]. These tissue explants are frequently used as an initial culture model to test newly developed biomaterials for neural engineering purposes. We used DRG in this study to determine the neural tissue response to surface nanotopography on electrospun fibers. All animal procedures in this study strictly adhered to the NIH Guidelines for the Care and Use of Laboratory Animals and were approved by the Institutional Animal Care and Use Committee at Rensselaer Polytechnic Institute. Postnatal day 2 (P2) Sprague-Dawley rats were purchased from Taconic Biosciences (Rensselaer, NY). Rats were euthanized via rapid decapitation and spinal columns were isolated and kept in ice-cold Hank’s Balanced Salt Solution (HBSS, Thermo Fisher, Cat. No. 14025134) for the duration of DRG isolation. Each spinal column was cut down the midline of the dorsal and ventral aspects using vannas scissors. We then removed DRG from each half of the spinal column using jeweler’s forceps. We excluded all DRG from the sacral and cervical spine, and pooled only DRG from the lumbar and thoracic spine in ice-cold Ham’s F12 nutrient mixture (Thermo Fisher, Cat. No. 11765054) until dissociation or culture onto electrospun fibers. DRG from the sacral and cervical spine are smaller than DRG from the lumbar and thoracic spine in neonatal rats. Thus, we excluded these DRG to prevent DRG size from affecting our experimental outcomes.

#### DRG dissociation

To expand upon our findings with DRG explants, we also cultured individual neurons onto the various fiber types to explore the single cell response to electrospun fiber surface nanotopography and determine if individual neurons respond similarly to neural tissue explants. Individual primary neurons were obtained by dissociating DRG. DRG were incubated for 50 minutes at 37°C in a buffer containing 0.1% Trypsin (Corning, Cat. No. 25-054-C1) and 1 mg/mL collagenase A (Sigma, Cat. No. C9891) in PBS. The microfuge tube containing DRG was inverted every 10 minutes to mix the contents and improve enzyme interaction with tissue. We then centrifuged DRG at 300 g for 5 minutes, removed the supernatant from the DRG pellet, and added 0.1% Trypsin in PBS to the DRG for an additional 10 minutes. Neuron media containing neurobasal media (Thermo Fisher, Cat. No. 12348017) supplemented with 50 ng/mL nerve growth factor (NGF, Thermo Fisher, Cat. No. 13257019), 1% Pen/Strep (Thermo Fisher, Cat. No. 15140–122), and 1% B27 media supplement (Thermo Fisher, Cat. No. 17504044) was then added to the incubating DRG (3:1 ratio of neuron media to trypsin solution to halt trypsinization) and the DRG were centrifuged again at 300 g for 5 minutes. We then aspirated the supernatant, raised the cells in neuron media, and gently triturated prior to counting cells and culturing onto electrospun fibers.

#### Electrospun fiber scaffold preparation for cell culture

Electrospun fibers were sterilized via ethylene oxide exposure in an Anprolene An74i tabletop sterilizer (Andersen Products, Haw River, NC). After sterilization, fibers were held in a sterile cell culture hood for three days to offgas any residual ethylene oxide. Prior to whole DRG culture, a subset of scaffolds were coated with 50 μg/mL laminin (Thermo Fisher, Cat. No. 23017015) in DI water for three hours. All fiber scaffolds for dissociated DRG culture were coated with laminin as individual neurons did not adhere to uncoated fibers. After laminin coating, scaffolds were washed 3x with sterile DI water, and all water was aspirated prior to DRG culture.

#### Whole DRG or dissociated DRG neuron culture onto electrospun fibers

Scaffolds for whole DRG were placed in 12-well plates, and submerged in 500 μL of neuron media. Whole DRG were then seeded onto smooth, pitted, and divoted fibers, either with or without laminin coating, by carefully picking up DRG with forceps and placing them onto the scaffolds. DRG were harvested from at least three different animals, and seeded onto at least three independently fabricated fiber scaffolds to conduct these experiments in biological and biomaterial triplicate. We were careful to place DRG near the center of each electrospun fiber scaffold to avoid neurites extending off the edge of the scaffolds. Samples were then moved to a cell culture incubator, maintained at 37°C and 5% CO_2_, and allowed to attach for 12 hours. After 12 hours, an additional 500 μL of neuron media was added to each DRG sample, and DRG were grown in the incubator for an additional four days prior to fixation.

Dissociated neurons were counted using a hemocytometer and seeded onto laminin coated electrospun fiber scaffolds at a seeding density of 100 cells/mm^2^. Similar to DRG culture, dissociated neurons were harvested from at least three different animals, and seeded onto at least three independently fabricated fiber scaffolds to conduct these experiments in biological and technical triplicate. This low seeding density was chosen so we could image and analyze the morphology of individual neurons. Cells were first suspended and triturated in 1 mL of neuron media prior to seeding on scaffolds. Dissociated neurons were grown in the cell culture incubator for 12 hours prior to fixation. Dissociated neurons were cultured for less time than whole DRG because they grew along the fibers more quickly. After growing for 24 hours on fibers, the growth of most neurons was limited by scaffold geometry. Thus, we chose to analyze dissociated neurons after 12 hours in culture.

### DRG fixation and immunocytochemistry

Whole DRG were fixed in a 4% paraformaldehyde (Electron Microscopy Sciences, Cat. No. 15710) solution in PBS for 15 minutes followed by three PBS washes. DRG were then incubated in a blocking solution containing 5% bovine serum albumin (BSA, Sigma, Cat. No. A9647) and 0.01% Triton X-100 (Sigma, Cat. No. T8787) in PBS at room temperature for 1 hour. The blocking solution was removed and DRG were incubated at 4°C overnight in a solution containing 5% BSA, 0.1% TWEEN-20, and a 1:500 dilution of RT-97 primary antibody (DSHB, Iowa City, Iowa), which stains against neurofilament. After primary antibody incubation, DRG were washed three times with PBS. A secondary antibody solution containing Alexa-Fluor donkey anti-mouse 488 (1:1000 dilution, Life Technologies Cat. No. R37114) was added to DRG for two hours at room temperature. DRG were washed three more times and held in PBS at 4°C until being imaged.

Dissociated neurons were fixed in a 4% paraformaldehyde solution in PBS for 15 minutes. Scaffolds were then washed three times in PBS and cells were blocked with 0.01% Triton X-100, 5% BSA in tris-buffered saline (TBS, Sigma, Cat. No. 28376) for 15 minutes at room temperature. Blocking buffer was then removed and cells were incubated overnight at 4°C in a solution containing 0.1% TWEEN-20 (Sigma, Cat. No. P1369), 5% BSA, and 1:500 RT-97 primary antibody in TBS. Scaffolds were washed three times with TBS, then incubated at room temperature for 1 hour in a solution containing donkey anti-mouse AlexaFluor 594 (1:1000 dilution, Life Technologies, Cat. No. A21203), 5% BSA, and 0.1% TWEEN-20 in TBS. After the incubation was complete, cells were washed three times in TBS and held in TBS at 4°C until being imaged. All cells were imaged using an inverted Olympus IX-81 spinning disc confocal microscope (Olympus, Tokyo, Japan). Confocal images were processed in FIJI to obtain 2D maximum intensity projections. Background was then subtracted from each image using a rolling ball algorithm with a radius of 40.0 pixels. Images were then stitched in Adobe Photoshop CS2 Version 9.0.

### Analysis of whole DRG morphology

To assess differences in DRG morphology among fibers with different surface topographies, we quantified three aspects of DRG morphology–neurite length, area of neurite coverage, and DRG aspect ratio. To quantify neurite length, the five longest neurites on each side of the DRG body were measured. Neurites were measured from the tip of the neurite to the perimeter of the DRG body using FIJI. To measure the area of neurite coverage, the perimeter of the DRG body was outlined in FIJI and measured to determine the area. Subsequently, the total area of the DRG and the area of the space between neurites were measured. The neurite area was determined by subtracting the area of the spaces between neurites and the area of the DRG body from the total area. We also characterized the aspect ratio of DRG cultured onto fibers by drawing a line that spanned the longest axis of the DRG (from the tip of the longest neurite on one side of the DRG body to the tip of the longest neurite on the opposite side), and dividing the length of this line by the length of a line that spanned the DRG in a perpendicular direction at its widest point. Anywhere from 6–12 DRG were analyzed on each fiber type (N = 6–12). Numbers varied due to some DRG not adhering to fibers, or getting washed away during immunocytochemistry staining procedures.

### Analysis of dissociated neuron morphology

Fluorescent images of dissociated neurons were analyzed using Neurolucida software (MBF Biosciences). Neurite outgrowth from individual neurons was traced semi-automatically. All individual neurons that did not overlap with neighboring neurons were traced. All traces were then processed using Neurolucida Explorer software to quantify values for the longest neurite, total neurite length, and the number of branch points on every neuron, to help discern differences between neurons growing on the different fiber types.

### Statistical analysis

Statistical analyses were performed using JMP IN software (Release 8.0.1; SAS, Cary, NC). Statistical significance of each result quantified in this study was determined via one-way ANOVA, followed by a post-hoc Student’s t-test to perform pairwise comparisons of each measured dependent variable (p<0.05). A Brown-Forsythe Test (p<0.05) was used to analyze the variances in alignment between the different electrospun fiber types.

## Results

### Electrospun fiber surface nanotopography and morphological properties

Electrospinning parameters were adjusted in this study to obtain three different types of fibers (Table 1), each with distinct surface nanotopography. Fibers that were electrospun at low relative humidity (<21%), and without DMSO (a non-solvent of PLLA) had smooth surfaces (Fig 1A). Fibers that were electrospun at a higher relative humidity (28–32%) with 1.8% w DMSO/w PLLA added into the electrospinning solution had well-defined surface depressions, hereby referred to as pits (Fig 1B). Fibers that were electrospun at high humidity (28–32%) without the addition of DMSO possessed a wrinkled surface without distinct surface depressions (Fig 1C). These fibers are referred to in this study as divoted fibers.

It was important to ensure that fiber alignment, diameter, and collection density were consistent among the three different fiber types to isolate fiber surface nanotopography as a variable that might affect DRG or dissociated neurons in culture. We first measured fiber alignment and found that all fibers of each different fiber type were highly aligned. Smooth, pitted, and divoted fibers were aligned within 6°, 12°, and 9° of mean fiber alignment, respectively (Fig 1D–1F). The diameter of smooth fibers (2.19 ± 0.19 μm) was not statistically different than the diameter of pitted (2.31 ± 0.06 μm, p = 0.485) or divoted fibers (2.44 ± 0.07 μm, p = 0.096) (Fig 1E). Lastly, we analyzed fiber collection density to ensure that cells cultured on each fiber type were exposed to scaffolds with similar fiber coverage. Smooth fibers collected on PLLA films at an average density of 362.57 ± 7.2 fibers/mm, which was not statistically different compared to the collection density of pitted (338.68 ± 20.98 fibers/mm, p = 0.248) or divoted fibers (342.41 ± 17.38 fibers/mm, p = 0.348) (Fig 1H). Because we observed differences in fiber diameter, although insignificant, it was important to ensure that these differences were not exacerbated by differences in fiber collection density to significantly alter the micro-scale of each scaffold that cells experienced in culture. Thus, we calculated the percent of fiber coverage for each fiber type (Fig 1I). Smooth fibers covered 79.14 ± 5.4% of the scaffold surface, which was not statistically significant compared to pitted (78.08 ± 3.54%, p = 0.946) or divoted (83.5 ± 2.91%, p = 0.443) fiber coverage. Thus, we validated that our fibers were morphologically similar on the micro-scale, which allowed us to isolate surface nanotopography as an independent variable for DRG and dissociated neuron culture experiments.

### Electrospun fiber surface area

**Fig 2:**
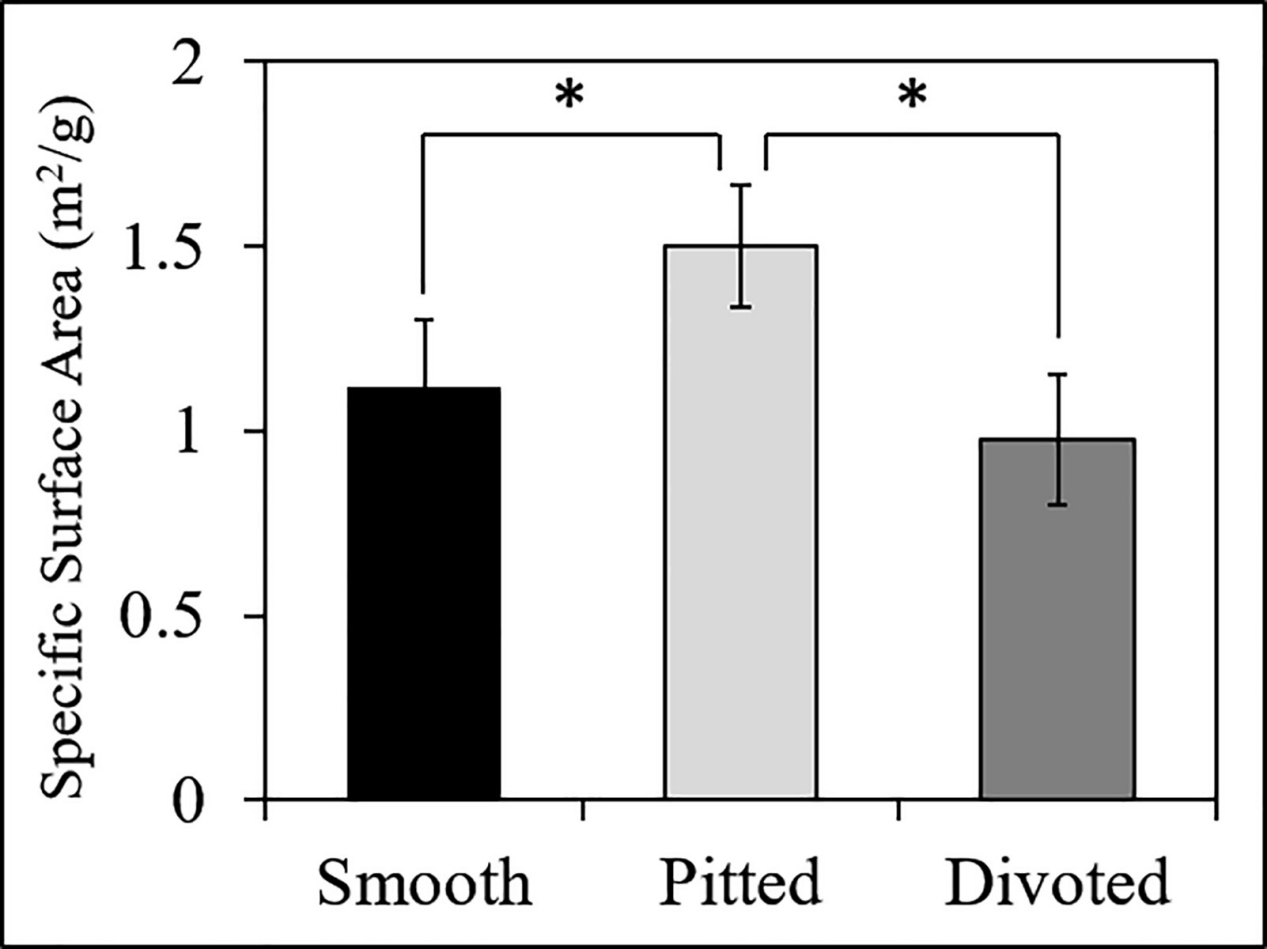
BET surface area analysis of smooth, pitted, and divoted electrospun fibers. The specific surface area of each electrospun fiber type was quantified via nitrogen gas physisorption. Statistical significance, determined by ANOVA and post-hoc Student’s t-test, is denoted by *(p<0.05). (Each type of electrospun fiber was analyzed in triplicate (N = 3), data are presented as mean ± standard deviation).

BET surface area analysis was conducted to quantify changes in specific surface area between the three electrospun fiber types used in this study. Fig 2 shows that the specific surface area of pitted fibers (1.5 ± 0.2 m^2^/g) was significantly higher than the specific surface areas of both smooth (1.1 ± 0.2 m^2^/g, p = 0.012) and divoted (0.9 ± 0.2 m^2^/g, p = 0.001) fibers. The difference between smooth and divoted fibers was not statistically significant.

### DRG neurite outgrowth

**Fig 3:**
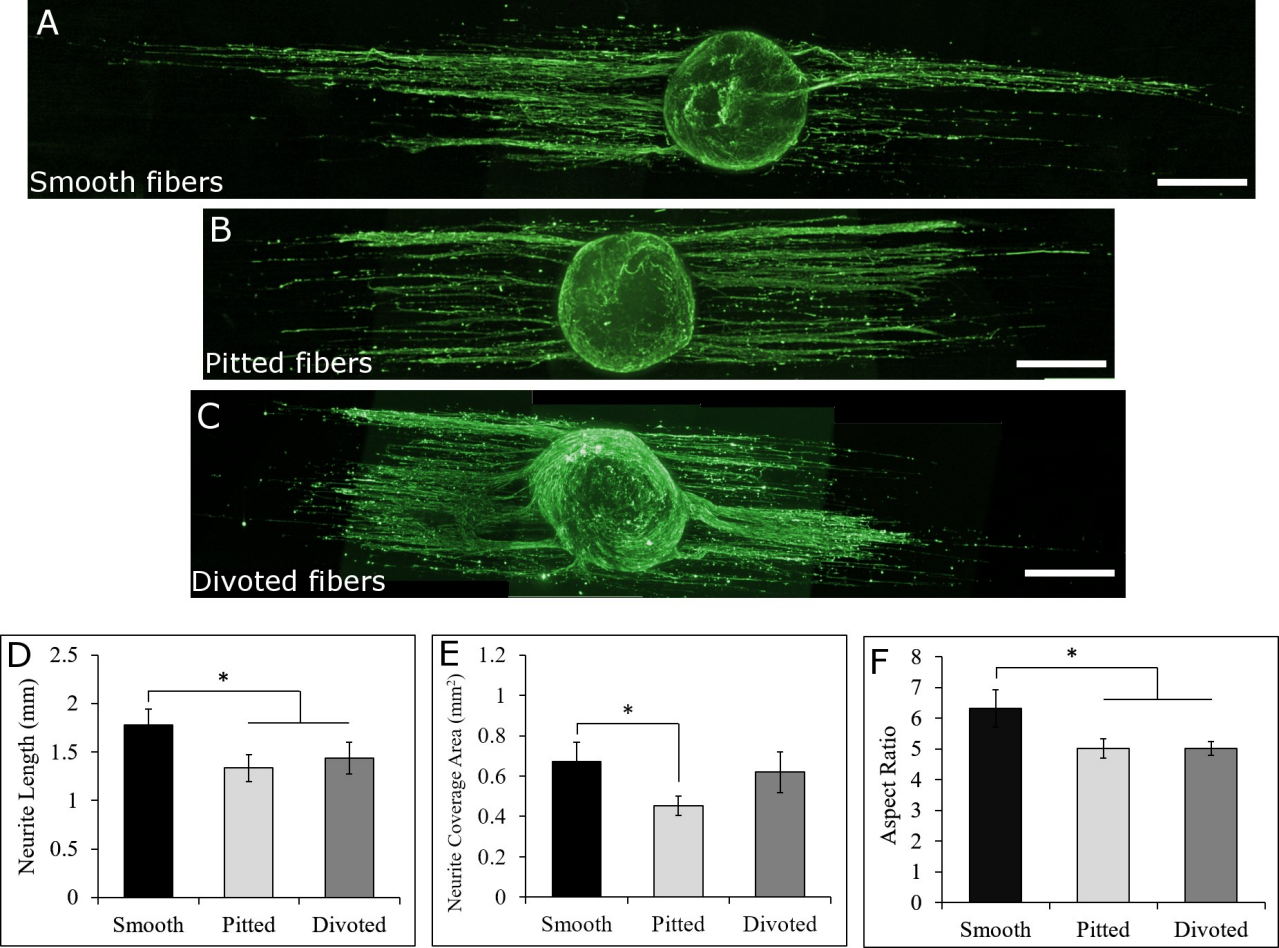
Whole DRG cultured on to uncoated smooth, pitted, and divoted fibers. DRG on smooth (A), pitted (B), and divoted (C) fibers responded differently to the various surface nanotopographical features on the fibers (400 μm scale bars). Neurite length (D) and coverage area (E) were quantified along with DRG aspect ratio to compare morphological differences between DRG on the different fiber types. Electrospun fibers (not shown) are oriented horizontally in all whole DRG images. Statistical significance between values in D-F was determined via ANOVA and post-hoc Student’s t-test (p<0.05). (For DRG on smooth fibers N = 8, on pitted fibers N = 7, and on divoted fibers N = 6, data are presented as mean ± standard error of means).

Whole DRG were cultured onto uncoated smooth (Fig 3A), pitted (Fig 3B), and divoted fibers (Fig 3C) for 4 days prior to fixation, imaging, and analysis. Based on previous studies in the Gilbert lab that showed macrophages and astrocytes exhibited a more elongated morphology on smooth fibers compared to fibers with surface topography [20,21], we hypothesized that DRG cultured onto pitted and divoted fibers would extend shorter neurites than DRG cultured onto smooth fibers. Fig 3D shows that neurites extended significantly farther from DRG cultured onto smooth electrospun fibers (1.77 ± 0.17 mm) compared to pitted (1.33 ± 0.1 mm, p = .009) and divoted fibers (1.43 ± 0.17 mm, p = 0.035). This finding supported our hypothesis that surface topography would decrease neurite extension. Further, Fig 3E shows that DRG neurite coverage was significantly greater on smooth fibers (0.67 ± 0.10 mm^2^) compared to pitted fibers (0.45 ± 0.05 mm^2^, p = 0.048) but not compared to divoted fibers (0.62 ± 0.10 mm^2^, p = 0.675). This further supported our hypothesis that surface topography would decrease neurite extension from DRG. Lastly, we characterized DRG aspect ratio (Fig 3F), which is the ratio of a DRG’s length to its width. The aspect ratio of DRG cultured onto smooth fibers (6.32 ± 0.61) was significantly greater than the aspect ratio of DRG on pitted (5.02 ± 0.31, p = 0.032) or divoted fibers (5.01 ± 0.22, p = 0.039). This aligns with the findings in Fig 3D, that DRG grow longer on uncoated smooth fibers compared to uncoated pitted or divoted fibers. This finding also shows that the decrease in neurite extension on pitted and divoted fibers is not correlated to an increase in perpendicular neurite outgrowth from the DRG body.

**Fig 4:**
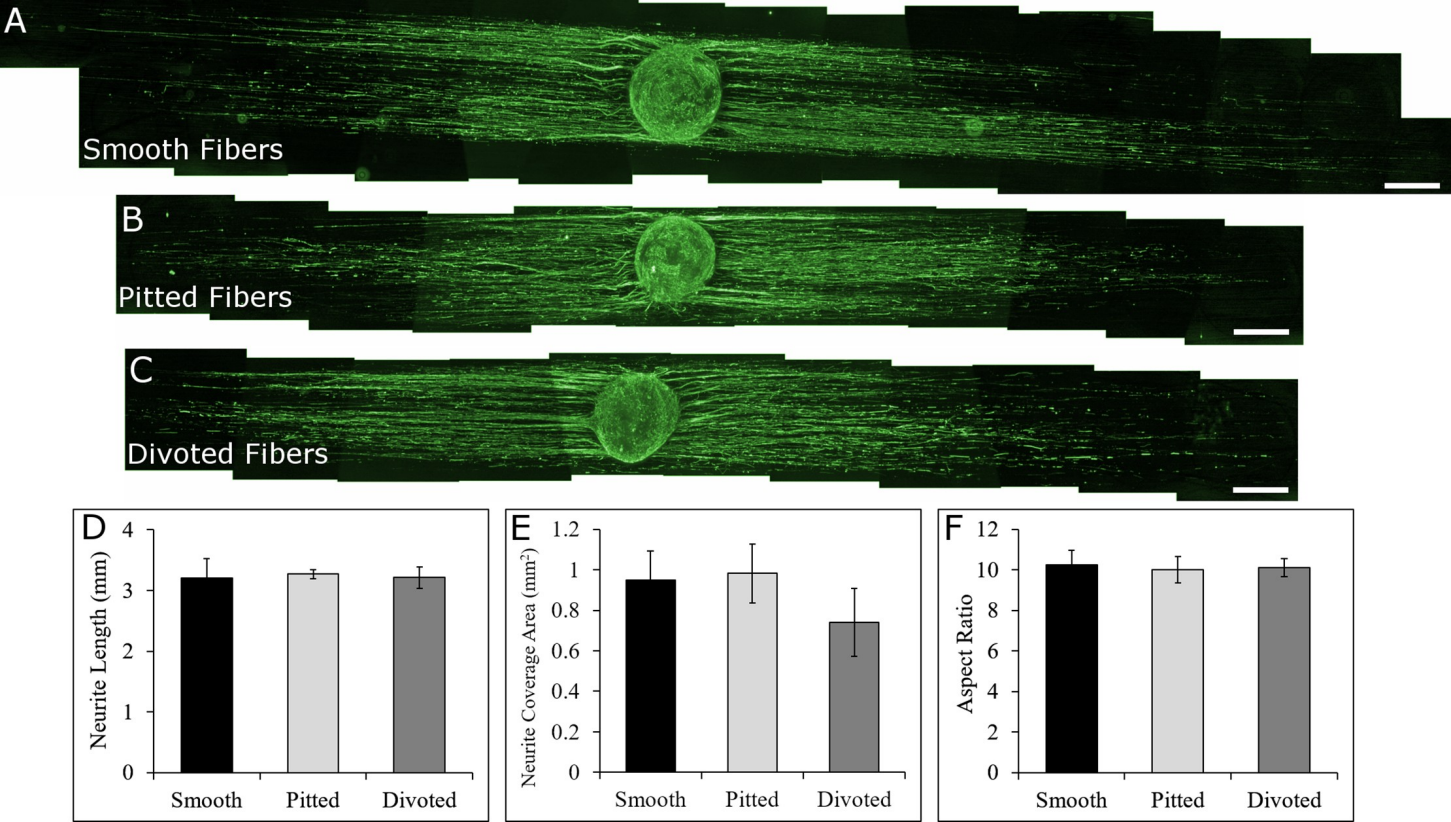
Whole DRG cultured on to laminin-coated smooth, pitted, and divoted fibers. DRG on laminin-coated smooth (A), pitted (B), and divoted (C) fibers responded similarly to the various surface nanotopographical features on the fibers after coating the fibers with laminin prior to culture (400 μm scale bars). Neurite length (D) and coverage area (E) were quantified along with DRG aspect ratio to compare morphological differences between DRG on the different fiber types. Electrospun fibers (not shown) are oriented horizontally in all whole DRG images. Statistical significance between values in D-F was determined via ANOVA and post-hoc Student’s t-test (p<0.05). (For DRG on smooth fibers N = 8, on pitted fibers N = 8, and on divoted fibers N = 9, data are presented as mean ± standard error of means).

After observing significant decreases in neurite outgrowth from DRG cultured onto pitted and divoted fibers, we hypothesized that laminin coating would rescue the nanotopography-induced decreases in neurite extension observed on uncoated fibers. Whole DRG were cultured onto laminin-coated smooth (Fig 4A), pitted (Fig 4B), and divoted fibers (Fig 4C) for the same amount of time as DRG were cultured onto uncoated fibers. Fig 4D shows that the length of neurites extending from DRG on smooth fibers (3.20 ± 0.32 mm) was not statistically significant compared to neurite extension from DRG cultured onto laminin coated pitted (3.27 ± 0.08 mm, p = 0.748) or divoted fibers (3.22 ± 0.18 mm, p = 0.951). Further, neurite coverage area (Fig 4E) in DRG cultured onto smooth fibers (0.95 ± 0.14 mm^2^) was not statistically different than the neurite coverage area on pitted (0.98 ± 0.15 mm^2^, p = 0.887) or divoted fibers (0.74 ± 0.17 mm^2^, p = 0.328). Lastly, the DRG aspect ratio (Fig 4F) was not impacted by fiber surface nanotopography after coating the fibers with laminin. The aspect ratio of DRG cultured onto laminin coated smooth fibers (10.24 ± 0.72) was not statistically significant compared to the aspect ratios of DRG cultured onto pitted (10.00 ± 0.65, p = 0.802) or divoted fibers (10.12 ± 0.46, p = 0.889). These findings validated our hypothesis that laminin coating would rescue any differences that we observed with DRG on uncoated fibers.

### Dissociated neuron morphology

After observing that laminin coating enabled neurites to extend similarly on all fiber surfaces, we explored whether or not surface nanotopography would impact dissociated neuron morphology. We used this approach to determine if neurons in tissue explants (DRG) and individual neurons responded similarly to the same materials, and also to elucidate how dissociated neurons would respond to electrospun fibers with surface topography. During this study, dissociated neurons did not adhere to fibers that were not coated with laminin, so neurons were only cultured on laminin coated fibers.

**Fig 5:**
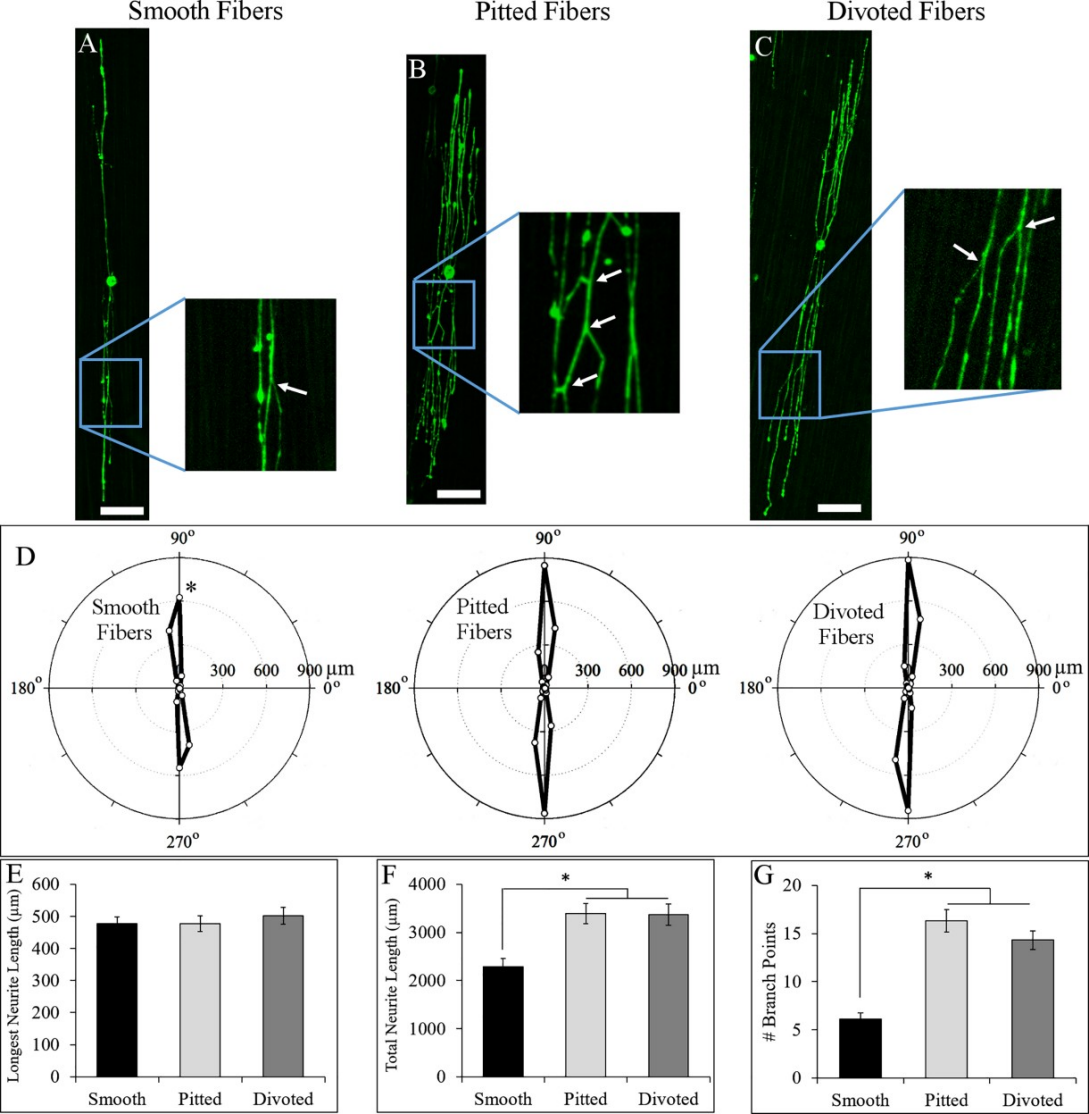
Dissociated neurons cultured on to laminin-coated smooth, pitted, and divoted fibers. Neurons on smooth (A), pitted (B), and divoted (C) fibers responded differently to the various surface nanotopographical features on the fibers despite being pre-coated with laminin (100 μm scale bars, white arrows highlight neurite branching points). Polar histograms of mean neurite length and directionality show qualitative differences between neurons on the different fiber types (D). Longest neurite length (E), total neurite length (F), and the number of branch points per neuron (G) were quantified to compare morphological differences between neurons on the different fiber types. Electrospun fibers (not shown) are oriented vertically in all neuron images. Statistical significance between values in D-G was determined via ANOVA and post-hoc Student’s t-test (p<0.05). (N = 46 for neurons on smooth fibers, 48 for neurons on pitted fibers, and 45 for neurons on divoted fibers, data are presented as mean ± standard error of means).

We hypothesized that dissociated neurons would respond to laminin-coated fibers in the same way that whole DRG did–without any observable significant differences between neurons cultured on the different fiber types. Fig 5A–5C show representative, dissociated neurons on smooth, pitted, and divoted fibers, respectively. Qualitatively, neurons on pitted and divoted fibers appeared to be broader, with more branching neurites than neurons on smooth fibers. Zoomed in images of neurons on each fiber type highlight the presence of neurite branch points (highlighted by white arrows in Fig 5A–5C). Polar histograms were created to represent the average neurite extension of all neurons that were imaged on each fiber type (Fig 5D). Polar histograms show that all fiber types promoted neurite extension along the orientation of fibers, and surface nanotopography did not impact the major direction of neurite extension. The average distance that neurites extended to, however, increased significantly on pitted and divoted fibers.

Dissociated neuron morphology was analyzed by tracing all neurites on a given neuron then analyzing the trace with Neurolucida software. This analysis showed that the longest neurite extending from dissociated neurons cultured on all three laminin-coated fiber types extended distances from the soma that were not statistically different (Fig 5G). The average lengths of the longest neurite on smooth fibers was 476.3 ± 22.6 μm, which was not statistically significant compared to pitted (476.4 ± 24.3 μm, p = 0.997) or divoted fibers (501.7 ± 26.0 μm, p = 0.464). Fig 5H, however, shows that dissociated neurons on laminin-coated pitted and divoted fibers exhibited significant increases in total neurite length–a measure of all lengths of all neurites extending from a given neuron, including all branching neurites. The average total length of all neurites on smooth, laminin-coated fibers was 2283.2 ± 170.2 μm per neuron. The average total length of all neurites on pitted or divoted laminin-coated fibers was 3389.5 ± 208.3 μm (p = 0.0003 compared to smooth fibers) and 3364.8 ± 221.6 μm (p = 0.0005 compared to smooth fibers) per neuron, respectively. We attributed this increase in total neurite length on pitted and divoted fibers to the increased branching that these neurons exhibited (Fig 5I). Neurons on laminin-coated, smooth fibers had an average of 6.1 ± 0.6 branch points per neuron. In comparison to neurons cultured onto smooth fibers, neurons on laminin-coated, pitted and divoted fibers had 16.3 ± 1.1 (p<0.0001) and 14.3 ± 1.0 (p<0.0001) branch points per neuron. Thus, we rejected our hypothesis that fiber surface topography would not have a significant impact on dissociated neuron morphology.

## Discussion

In this study, we explored the effects of electrospun fiber surface nanotopography on neurons in culture. Our major findings were: 1) Pitted fibers possessed a significantly greater specific surface area than smooth and divoted fibers, 2) Uncoated electrospun fibers with either pitted or divoted surface nanotopography hindered neurite extension from a DRG explant when compared to smooth fibers, and coating fibers with laminin rescued these decreases in neurite extension, and 3) Laminin-coated electrospun fibers with pitted or divoted surfaces caused dissociated neurons to develop a more branched structure with increased secondary neurite growth compared to neurons grown on smooth fibers. These findings are important in the field of biomaterials and tissue engineering as they show the significant impact that nano-scale surface modifications can have on adhering neurons. Thus, our findings may inform researchers implementing electrospun fiber scaffolds for *in vivo* tissue engineering applications.

In recent years, the Gilbert laboratory has developed methods to control the formation of nano-scale surface depressions on the surface of electrospun fibers [19–21]. Researchers in the Gilbert laboratory cultured primary bone marrow macrophages onto electrospun fibers with smooth surfaces or with surface depressions. Macrophages on fibers with surface depressions exhibited a longer and thinner morphology and increased production of the anti-inflammatory cytokine IL-12, compared to macrophages on smooth fibers [19]. In a more recent study, researchers cultured primary astrocytes (derived from rat spinal cords or cortices), and a coculture of either astrocyte type and a DRG explant onto smooth, pitted, and divoted fibers. This study elucidated how fiber surface nanotopography affects astrocyte biology, and astrocyte mediated neurite extension from DRG [21]. Cortical astrocytes elongated significantly further along smooth fibers compared to fibers with pitted or divoted surfaces. Spinal cord astrocytes on the various fiber types did not show significant differences in morphology. Spinal cord-derived astrocytes on smooth fibers did, however, elicit nearly a two-fold increase in DRG neurite extension compared to spinal cord-derived astrocytes cultured onto fibers with surface nanotopography [21]. This finding coincides with our observation that DRG on uncoated, smooth fibers extended significantly longer neurites than DRG on uncoated, pitted or divoted fibers. Until now, however, no study has thoroughly explored the effects that nanoscale surface depressions on electrospun fibers have solely on primary neurons in culture.

Prior to neuron culture, we fine-tuned our electrospinning parameters to ensure that the physical properties of smooth, pitted, and divoted fibers were statistically similar, with the exception of surface nanotopography. This was imperative to isolate the effects of surface nanotopography on neurons in culture. It was also imperative to produce fibers with consistent diameters when analyzing fiber specific surface area. The surface area of a fiber depends on the fiber’s radius and the square of the fiber’s radius. Thus, controlling for diameter was necessary to ensure that changes in specific surface area were attributable to differences in surface nanotopography and not fiber diameter.

Using BET analysis, Zhang et al. observed a nearly 2.5-fold increase in the specific surface area of gelatin/PCL fibers following removal of gelatin from the fibers through leaching [24]. The results from the study by Zhang and colleagues were used to generate our hypothesis that inducing a pitted or divoted surface on PLLA fibers would significantly increase the fiber specific surface area. The BET data gathered after analyzing pitted fibers supported our hypothesis that surface nanotopography increases the specific surface area. The hypothesis was rejected after analyzing data gathered from BET analysis of the divoted fibers, where the specific surface area decreased by 18% when compared to the smooth fibers. We suspect that divoted fibers have a higher crystallinity than smooth fibers since the divoted fibers were created in humid air. Under humid conditions, PLLA hydrolyzes and the remaining small degradation products can result in increased crystallinity [25,26]. A PLLA fiber with greater crystallinity would contain more PLLA and have a higher polymer density than a fiber with the same diameter and lesser crystallinity. This increase in polymer density in the divoted fibers may overshadow the increased fiber surface area. Although pitted fibers were also created in humid air, an increase in fiber density resulting from PLLA crystallization in humid air may have been negated by the presence of DMSO, which created pits in the fibers. Future studies will analyze how fiber surface topography influences polymer crystallinity.

After thoroughly analyzing fiber morphology and surface area, we cultured whole DRG onto uncoated, smooth, pitted, and divoted fibers. Only two studies, to our knowledge, have analyzed the effects of electrospun fiber surface topography on neurons. A 2009 study by Lee et al. studied the effects of surface nanotopography *in vitro* with PC12 cells and primary rat hippocampal neurons [11]. Lee and colleagues coated electrospun poly(lactic-co-glycolic acid) fibers with polypyrrole, which created nano-scale surface protrusions on the fibers. The surface protrusions did not significantly impact neurite extension from PC12 cells or primary hippocampal neurons, but the chemical effects of polypyrrole in this study were not controlled for and may have confounded these results. In a more recent study, Ziemba and colleagues cultured embryonic chick DRG onto smooth electrospun fibers and onto electrospun fibers with surface protrusions and depressions. This study showed that DRG on fibers with pits and protrusions extended neurites approximately 65% farther than DRG cultured onto smooth fibers [13]. This study, however, did not control for fiber diameter, and the average diameter of fibers with pits and protrusions (2.61 ± 0.67 μm) was significantly larger than the diameter of smooth fibers (1.75 ± 0.74 μm) used in that study. Thus, the effects of electrospun fiber surface nanotopography on neurons remained to be explicitly studied. In the current study, we observed that DRG neurite extension was significantly shorter on both pitted and divoted fibers, compared to smooth fibers (when fibers were uncoated). This result disagrees with the findings by Ziemba and colleagues that also studied uncoated fibers, and suggests that the increased neurite extension observed in that study may not be the direct result of differences in surface topography on the different fiber types.

In a 2014 study, Xie et al. thoroughly studied the effects of electrospun nanofiber surface coatings on the directionality and length of DRG neurite extension [15]. DRG cultured onto uncoated, aligned nanofibers extended neurites both along the axis of fiber alignment, and perpendicular to the axis of fiber alignment. Neurite outgrowth perpendicular to the long axis of the aligned fibers was likely due to neurites recognizing nanoscale void spaces between individual fibers for contact guidance and using these voids to direct their perpendicular outgrowth. When nanofibers are packed closely together, cells can extend perpendicular to the axis of fiber orientation if the space between fibers is small (also on the nanoscale), rather than growing along the axis of fiber orientation [27]. Xie and colleagues also coated the nanofibrous scaffolds with poly(L-Lysine) (PLL) or PLL followed by different concentrations of laminin. Fluorescent images of DRG on each scaffold showed that neurite extension in the direction of fiber alignment increased significantly with increases in laminin concentration, whereas PLL coating alone did not significantly alter neurite extension compared to uncoated controls (although this comparison was not quantified). Thus, it appeared that laminin coating of electrospun fibers allows neurites to largely ignore contact guidance created by the voids between individual fibers and focuses neurite extension along individual fibers more successfully. While the guidance cues presented by Xie and colleagues were smooth nanofibers and not microfibers with nanotopography as presented here, nanotopography on microfibers may influence neurite outgrowth in a similar manner. Due to the difference in fiber diameter between the current study and the study by Xie et al., the voids between fibers were likely comparable in size to the nanotopographical features on the microfibers used herein.

Results from the Xie et. al. study where neurite outgrowth was perpendicular to the aligned, uncoated nanofibers and laminin coating counteracted the perpendicular outgrowth motivated our hypothesis that coating divoted or pitted microfibers with laminin would better direct neurites along microfiber topographies. Whole DRG cultured onto laminin-coated smooth, pitted, and divoted fibers all exhibited similar morphologies in terms of neurite extension, coverage area, and DRG aspect ratio. These results supported our hypothesis that laminin coating would rescue deficits in neurite extension caused by surface nanotopography. It is important to note that other proteins, in addition to laminin, may also help extending neurites navigate through surface nanotopography. However, no other study has examined the ability of another extracellular matrix protein to improve neurite outgrowth along nanotopographical features. But, the ability of different extracellular matrix proteins to more robustly promote neurite extension along aligned electrospun fibers without nanotopography is known. A recent study by Soliman et al. demonstrated that coating electrospun fibers with the extracellular matrix protein fibronectin prior to neural culture increased neurite extension by nearly a factor of three after 72 hours in culture, when compared to uncoated fibers [28]. Even though the study by Soliman and colleagues used fibers without surface nanotopographical features, the results clearly demonstrate the ability of extracellular matrix protein coatings (besides laminin coatings) to enhance axonal regeneration on fibrous surfaces. After experimenting with whole DRG, we were still interested in how these results might translate to dissociated neurons.

As we mentioned previously, no studies have conducted a controlled, thorough investigation of the effects of fiber surface nanotopography on neurons. A recent study by Ventre et al., however, studied the effects of ultrasound on neuron morphology, and observed similar differences in neurite growth from neurons exposed to high intensity ultrasound [29]. Interestingly, Ventre and colleagues observed a 2-fold increase in neurite branching and a 2.8-fold increase in total neurite growth from neurons exposed to high-intensity ultrasound compared to unstimulated controls. The differences that we observed (2.7-fold increase in branching and 1.5-fold increase in total neurite length) by simply modifying fiber surface nanotopography were comparable to the Ventre study’s use of this promising neuromodulation therapy. While the two studies vary largely in approach, the similarity in their findings may speak to the therapeutic potential of utilizing growth substrate surface nanotopography to modulate the cellular response to biomaterial intervention.

A study by Voyiadjis et al. provides a potential explanation for how surface topography on fibers may lead to increased neurite branching [30]. Voyiadjis and colleagues showed that neurites that interacted with other neurites at shallow angles fasciculated into a bundle, while neurites interacting at steeper angles would not. This suggests that the nano-scale guidance cues on pitted and divoted fibers may coax branching neurites away from mean neurite alignment at an angle that is too steep to allow for fasciculation. This could explain the observed increase in total neurite length–neurites were not bundling together, but rather branching off and growing independently along fibers after branching. Whereas, on smooth fibers, neurites that branched off of the primary neurite tended to re-fasciculate a short distance after. Increased branching and defasciculation may lead to a higher potential to reinnervate muscles and increase neurotransmitter release/uptake near the terminal ends of neurites [31]. Other studies, however, suggest that robust defasciculation further away from the terminal ends of neurites can hinder motoneuron pathfinding and obstruct functional recovery after nervous system injury [32–34]. Thus, modifying electrospun fiber surface topography may cause beneficial changes in the neuron response, but more work should be done to better understand the implications of these findings.

Future research will explore the mechanisms by which nano-scale fiber surface modifications affect neurite growth and branching. A number of studies describe the interaction between the proteins axonin-1 and NgCAM as an important process to facilitate neurite fasciculation [35,36]. It will be interesting to research if surface depressions modulate expression of either of these proteins, or if the increased branching prevents axonin-1 and NgCAM on neighboring branches from interacting with each other. Further, there is strong evidence that expression of polysialic acid on neural cell adhesion molecules causes defasciculation of neurite bundles [37,38]. Thus, future studies should explore polysialic acid expression in neurons cultured onto the various fiber types.

## Conclusion

Aligned, electrospun fibers have demonstrated great potential in directing the regeneration of axons in the peripheral and central nervous systems. In this study, we found that unique nanotopography on the surfaces of aligned, electrospun fibers restricted the extension of neurites from whole dorsal root ganglia and that laminin coating on these surfaces counteracted this restriction. Using dissociated DRG cultures, we observed that neurites on laminin-coated divoted or pitted fibers were more branched, suggesting a potential mechanism by which surface nanotopography may influence neurite extension. In total, this study is the first to examine how neurite outgrowth is influenced by electrospun fiber surface nanotopography, and we observe that nanotopographies can increase total neurite growth and branching in the presence of an extracellular matrix coating. Future work will focus on determining intrinsic signaling pathways that may be involved in nanotopography-induced neurite branching.
